# Transient pre‐seizure inhibition of lateral hypothalamic orexin neurons: a novel possibility for seizure control

**DOI:** 10.1002/mco2.70070

**Published:** 2025-01-14

**Authors:** Yuang Gu, Yi Wang, Heming Cheng

**Affiliations:** ^1^ Key Laboratory of Neuropharmacology and Translational Medicine of Zhejiang Province Department of Neurology The First Affiliated Hospital of Zhejiang Chinese Medical University (Zhejiang Provincial Hospital of Chinese Medicine) School of Pharmaceutical Sciences Zhejiang Chinese Medical University Hangzhou China

1

A recent study published in *Nature Communications* revealed that the pre‐seizure activity of hypocretin/orexin neurons (HONs) in the lateral hypothalamus (LH) plays a pivotal role in determining the severity of subsequent seizures.^1^ The temporary inhibition of this activity has been shown to effectively reduce the intensity of seizures. This study offers novel insights into potential strategies for precise seizure prediction and the treatment of epilepsy.

Epilepsy, which is characterized by unpredictable recurrent seizures, is one of the most prevalent and severe neurological disorders. However, issues such as poor tolerance and adverse effects often impede the clinical efficacy of chronic anti‐seizure medications. HONs globally regulate brain excitability by releasing excitatory hypocretin/orexin peptides and glutamate, thereby playing crucial roles in pivotal physiological functions such as arousal and energy metabolism. Previous studies have demonstrated that the pharmacological suppression of HONs has promising anti‐seizure effects; however, it also leads to long‐term side effects. Therefore, it is essential to identify the specific period in which HON activity contributes to seizures and to implement precise interventions during this stage.

Initially, Li et al. used a mouse optokindling seizure model via the optogenetic stimulation of glutamatergic neurons in CA1 with 20 Hz, 10 s of laser stimulation. They reported that intraperitoneal administration of the orexin receptor antagonist SB‐334867 effectively reduced the susceptibility to and power of optokindling seizures without affecting seizure duration; however, it also notably decreased spontaneous locomotor activity in mice. Moreover, the intracerebroventricular injection of SB‐334867 was found to shorten the duration of different seizure stages in pentylenetetrazol kindling‐induced seizures,^2^ thus indicating distinct anti‐seizure properties of HONs across various seizure models.

Subsequently, Li et al. used calcium fiber photometry to monitor the activities of HONs during different epochs of optokindling seizures. Compared to the pre‐seizure period, there was a significant and pronounced increase in HON activity during seizures, thus indicating their involvement in seizure propagation. Interestingly, seizure power was found to be positively correlated with HON activity preceding the onset of seizures rather than during the seizure event itself. Furthermore, multivariate analysis provided additional support for the notion that pre‐seizure HON activity plays a more significant role in determining subsequent seizure intensity. These findings introduce a novel perspective for seizure prediction by monitoring HON activity. However, it should be noted that hippocampal optokindling seizures are specifically induced by optogenetic stimulation, which differs from the unpredictable recurrent seizures that are observed in clinical settings. Future investigations could involve kainic acid‐ or pilocarpine‐induced chronic seizure models to further validate the correlation between pre‐seizure HON activity and subsequent spontaneous seizure intensity (Figure 1).

**FIGURE 1:**
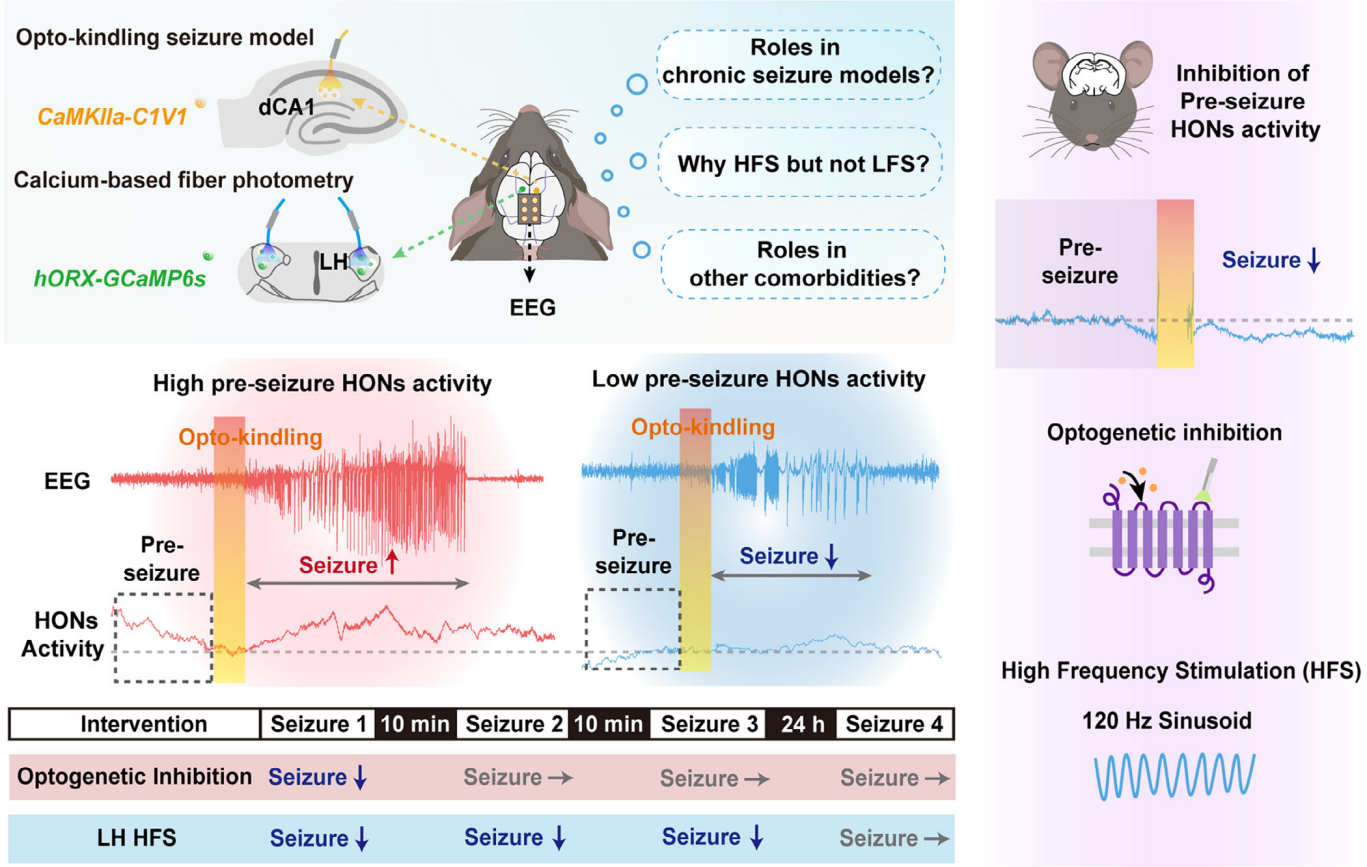
Calcium‐based fiber photometry combined with electroencephalogram (EEG) recording reveals a positive correlation between the pre‐seizure activity of hypocretin/orexin neurons (HONs) and subsequent optokindling seizures. Pre‐seizure optogenetic inhibition of HON activity results in transient anti‐seizure effects, whereas high‐frequency stimulation (HFS) of the lateral hypothalamus (LH) produces a prolonged effect. These findings offer novel insights for the development of seizure prediction and epilepsy treatment; however, several issues remain to be answered. Question 1: What is the role of pre‐seizure activity of HONs in chronic seizure models? Question 2: Why does HFS inhibit hyperactivity in HONs compared to low‐frequency stimulation (LFS)? Question 3: What is the role of HONs in other comorbidities associated with epilepsy?

To investigate the causal relationship between HON activity and seizure intensity, Li et al. used optogenetics to selectively inhibit bilateral LH HONs during specific time periods. They reported that the inhibition of HONs during seizures had no effect on seizure severity; however, the inhibition of HONs at 1 min prior to seizures significantly reduced the intensity, probability, and severity of subsequent seizures. Nevertheless, the anti‐seizure effect of pre‐seizure HON inhibition was transient, with a duration not exceeding 10 min. In summary, these results suggest that pre‐seizure HON activity contributes to subsequent seizures and that acute pre‐seizure inhibition of HON activity effectively suppresses subsequent seizures.

Emerging evidence suggests that deep brain stimulation (DBS) is an alternative therapeutic strategy for drug‐resistant epilepsy; however, its efficacy is closely associated with the stimulation target, employed parameters, and treatment epochs. To investigate the potential of the LH as a target for DBS in seizure treatment, Li et al. applied 1 min of sinusoid DBS (at either 2 Hz or 120 Hz) to the LH prior to seizures and simultaneously monitored HON activity. The results demonstrated that sinusoid high‐frequency (120 Hz) hypothalamic DBS (shhDBS) significantly suppressed HON activity and attenuated subsequent seizures, whereas low‐frequency DBS (2 Hz) did not have any effect. Additionally, in conjunction with the optogenetic modulation results, only pre‐seizure shhDBS effectively suppressed subsequent seizures, whereas DBS performed during seizures or with a latency of 10 s had no discernible effect. Interestingly, the therapeutic time window of shhDBS was prolonged compared to that of optogenetic modulation, as evidenced by the sustained effects observed even at 20 min after DBS. It should be noted that DBS is a nonselective technique; specifically, it can modulate both cells located in the LH and cells in upstream projecting or pathway fibers. Further investigations are warranted to elucidate their respective roles in epilepsy. Novel tools, including various neuromodulator and neuropeptide sensors and probes, may help with further verification.^3^ Another intriguing point with respect to DBS is the variability in the frequency of DBS in epilepsy across different brain regions. Previous studies have shown that high‐frequency stimulation (HFS) of the thalamus effectively reduces seizure frequency,^4^ whereas low‐frequency stimulation (LFS) targeting limbic system structures such as the entorhinal cortex, subiculum, and medial septum has an anti‐seizure effect.^5^ Some studies have suggested that HFS can induce long‐term potentiation, whereas LFS can induce long‐term depression. In this study, HFS of the LH (rather than LFS) successfully inhibited the hyperactivity of HONs. Further investigations are warranted to explore whether this observation is related to differences in electrophysiological characteristics among different brain regions.

Finally, Li et al. reported that acute shhDBS has no effect on the basic physiological functions of mice. Epilepsy is often comorbid with neuropsychiatric disorders, including anxiety, depression, cognitive impairments, and sleep rhythm disturbances. Previous research has shown that SB‐334867 can reverse anxiety‐like behaviors in a pentylenetetrazol model of seizures. Therefore, it is also worth investigating whether shhDBS can alleviate the comorbidities of epilepsy. Taken together, these findings suggest that acute shhDBS demonstrates significant promise as a potential anti‐seizure strategy without any side effects.

In summary, Li et al. reported that the pre‐seizure activity of HONs contributes to subsequent seizure intensity and that the inhibition of HON activity via optogenetics or shhDBS can effectively exert anti‐seizure effects, thereby providing a novel approach for the development of seizure prediction and epilepsy treatment.

## AUTHOR CONTRIBUTIONS

Heming Cheng and Yi Wang conceived the study and provided revisions and supervision. Yuang Gu conducted the literature research, drafted the manuscript, and created the figures. All of the authors have read and approved the final manuscript.

## CONFLICT OF INTEREST STATEMENT

The authors declare no conflicts of interest.

## Data Availability

Not Applicable.
